# Significantly Low Levels of IgG Antibodies Against Oncogenic Merkel Cell Polyomavirus in Sera From Females Affected by Spontaneous Abortion

**DOI:** 10.3389/fmicb.2021.789991

**Published:** 2021-12-14

**Authors:** Chiara Mazziotta, Giulia Pellielo, Mauro Tognon, Fernanda Martini, John Charles Rotondo

**Affiliations:** ^1^Department of Medical Sciences, University of Ferrara, Ferrara, Italy; ^2^Center for Studies on Gender Medicine, Department of Medical Sciences, University of Ferrara, Ferrara, Italy; ^3^Laboratory for Technologies of Advanced Therapies (LTTA), University of Ferrara, Ferrara, Italy

**Keywords:** spontaneous abortion, miscarriage, pregnancy, viral infection, Merkel cell polyomavirus, antibodies, IgG, immune system

## Abstract

Merkel cell polyomavirus (MCPyV) is a small DNA tumor virus ubiquitous in humans. MCPyV establishes a clinically asymptomatic lifelong infection in healthy immunocompetent individuals. Viral infections are considered to be risk factors for spontaneous abortion (SA), which is the most common adverse complication of pregnancy. The role of MCPyV in SA remains undetermined. Herein, the impact of MCPyV infection in females affected by SA was investigated. Specifically, an indirect enzyme-linked immunosorbent assay (ELISA) method with two linear synthetic peptides/mimotopes mimicking MCPyV antigens was used to investigate immunoglobulin G (IgG) antibodies against MCPyV in sera from 94 females affected by SA [mean ± standard deviation (SD) age 35 ± (6) years] and from 96 healthy females undergoing voluntary pregnancy interruption [VI, mean (±SD) age 32 ± (7) years]. MCPyV seroprevalence and serological profiles were analyzed. The overall prevalence of serum IgG antibodies against MCPyV was 35.1% (33/94) and 37.5% (36/96) in SA and VI females, respectively (*p* > 0.05). Notably, serological profile analyses indicated lower optical densities (ODs) in females with SA compared to those undergoing VI (*p* < 0.05), thus indicating a reduced IgG antibody response in SA females. Circulating IgGs were identified in sera from SA and VI females. Our immunological findings indicate that a relatively reduced fraction of pregnant females carry serum anti-MCPyV IgG antibodies, while SA females presented a more pronounced decrease in IgG antibody response to MCPyV. Although yet to be determined, this immunological decrease might prompt an increase in MCPyV multiplication events in females experiencing abortive events. The role of MCPyV in SA, if present, remains to be determined.

## Introduction

Merkel cell polyomavirus (MCPyV) is a small DNA tumor virus (Witkin et al., 2020). It is the main causative agent of Merkel cell carcinoma (MCC), which is a rare, but highly aggressive, non-melanoma skin cancer (Rotondo et al., 2017; Jin et al., 2019). MCPyV oncogenic activity is mediated by viral DNA integration into the host genome (Rotondo et al., 2017; Del Marmol and Lebbé, 2019; Pietropaolo et al., 2020), alongside the expression of two viral oncoproteins large T (LT) and small T (sT) antigens (Csoboz et al., 2020; Liu and You, 2020). Two additional MCPyV proteins are major capsid protein 1 (VP1) and minor capsid protein 2 (VP2), which present structural functions (Pietropaolo et al., 2020). VPs have previously been exploited as immunoantigens in studies focusing on the detection of immunoglobulin G (IgG) antibodies against MCPyV in humans (Touzé et al., 2010, 2011). Serological studies have indicated that MCPyV is ubiquitous in the healthy population, with varying rates reported as ranging from 60 to 80%, approximately (Carter et al., 2009; Kean et al., 2009; Pastrana et al., 2009; Faust et al., 2011; Tolstov et al., 2011; Touzé et al., 2011; Viscidi et al., 2011; Coursaget et al., 2013; Van Der Meijden et al., 2013; Šroller et al., 2014; Zhang et al., 2014; Vahabpour et al., 2016; Kamminga et al., 2018; Csoboz et al., 2020; Zhou et al., 2020; Mazziotta et al., 2021a,b). Initial exposure to MCPyV occurs early in life. Then, MCPyV establishes a lifelong, asymptomatic infection in healthy immunocompetent individuals (Hashida et al., 2016; Prezioso et al., 2019). However, in certain circumstances, such as during host immune system impairment, increased MCPyV replication levels/activity can occur, thereby leading to an increase in MCC occurrence (Rotondo et al., 2017; Tabachnick-Cherny et al., 2020; Decaprio, 2021).

Spontaneous abortion (SA) is the natural loss of pregnancy before the 20th week of gestation and represents the most common adverse complication in pregnancy. Approximately 10–20% of clinically recognized pregnancies end in a spontaneous loss of the embryo/fetus (Hertz-Picciotto and Samuels, 1988; American College of Obstetricians and Gynecologists Committee on Practice Bulletins—Gynecology, 2018).

Spontaneous abortion causes comprise genetic abnormalities in either partners, which may lead to abnormal chromosomal numbers or alterations (Eiben et al., 1990; Suzumori and Sugiura-Ogasawara, 2012; Dean et al., 2018). Additional SA causes include negative lifestyle factors, such as unhealthy diet/weight other than smoking, alcohol and drug abuse, and other factors, such as ethnicity, stress, and occupational/chemical exposures (De La Rochebrochard and Thonneau, 2002; Sopori, 2002; Lashen et al., 2004). Hormonal, anatomical, and autoimmune abnormalities, as well as male genetic/epigenetic factors, have also been identified (Toth et al., 2010; Rotondo et al., 2012, 2013, 2021a). Notably, although a variety of SA factors have been reported, other causes are yet to be determined (Miyaji et al., 2019; Fukuta et al., 2020). Indeed, nearly one-half of SA cases, defined as idiopathic, presents an undefined etiology (Jeve and Davies, 2014).

A growing number of studies have identified infectious agents as SA risk factors (Giakoumelou et al., 2016; Contini et al., 2018). Nearly 40% of SA cases are estimated to be linked to infectious agents, including viruses (Donders et al., 2000; Srinivas et al., 2006; Giakoumelou et al., 2016), while over 60% of females experience at least one infection during pregnancy (Collier et al., 2009). Viruses can therefore cause severe complications during pregnancy and are reported as associated with cases of stillbirth and preterm delivery, in addition to SA (Giakoumelou et al., 2016; Racicot and Mor, 2017).

Different human viruses, such as dengue, Zika, adeno- and adeno-associated viruses, human cytomegalovirus (HCMV), herpes simplex viruses 1 and 2 (HSV-1 and HSV-2), and the recently discovered severe acute respiratory syndrome coronavirus 2 (SARS-CoV-2), can potentially impact pregnancy outcome, ending, in the worst cases, in an SA event (Burton and Watson, 1997; Fisher et al., 2000; Giakoumelou et al., 2016; Azevedo et al., 2018; Carabali et al., 2018; de Freitas et al., 2018; Sayyadi-Dehno et al., 2019; Auriti et al., 2021; Rotondo et al., 2021b).

The limited amount of data available preclude robust conclusions on the clinical significance of polyomavirus (PyV) infections, including MCPyV, in SA from being made (Tagliapietra et al., 2019, 2020; Mazzoni et al., 2020). Footprints of viral DNAs from PyVs, including Simian virus 40 (SV40), JCPyV/BKPyV, and MCPyV, have been detected at low prevalence in umbilical cords, placenta, peripheral blood mononuclear cells (PBMCs), and/or chorionic villi from both pregnant and SA females (Sadeghi et al., 2010; Tagliapietra et al., 2019, 2020; Mazzoni et al., 2020). Immunological data have also indicated the presence of circulating IgGs against SV40, JCPyV, and BKPyV in the same study groups (Tagliapietra et al., 2019, 2020). A role in SA has been excluded for other PyVs, i.e., KIPyV and WUPyV (Sadeghi et al., 2010). Notably, until now, only one study has reported on the presence of circulating antibodies against MCPyV in pregnant females (Sadeghi et al., 2010), while MCPyV serology in females with SA is unknown.

The lack of immunological data in this field has raised the question of whether MCPyV infection might be linked to SA. To this purpose, we aimed to assess the MCPyV seroprevalence and serological profiles in two sets of sera belonging to females affected by SA, the case, and healthy females (HF) undergoing voluntary interruption of pregnancy (VI), as control.

## Materials and Methods

### Human Sera

Human sera were obtained from females who had experienced SA (*n* = 94), i.e., the case, and females undergoing VI (*n* = 96), i.e., the control. SA and VI sera were from our archive (Tognon et al., 2020). Samples were collected within 12 h from the abortion. Samples were stored at −80°C until testing, as reported previously (Mazziotta et al., 2021a,b). The mean ages [± standard deviation (SD)] of SA and VI groups were 35 ± 6 and 32 ± 7 years (*p* > 0.05), respectively. SA and VI inclusion criteria were (i) patients aged 18–42 years; (ii) gestational age within the first 12 weeks; and (iii) for the VI group, females selected according to Italian Law 194, article 6, comma B. Exclusion criteria were (i) severe hormonal or uterine dysregulations; (ii) immunosuppressive therapies known to cause SA events; (iii) genetic disorders; (iv) presence of infections, such as hepatitis B virus (HBV), human immunodeficiency virus (HIV), hepatitis C virus (HCV), and syphilis; (v) use of teratogenic drugs; and (vi) acquired/congenital immunodeficiency syndromes/diseases. Written informed consent was obtained from all subjects/patients according to the Declaration of Helsinki. The Ethical Committee of Ferrara, Italy, authorized the study (ID: 151078). In addition, immunological data from a set of sera belonging to a cohort of age-matched HF [*n* = 95, mean age ± SD, 34 ± (9) years] (*p* > 0.05), from our previous study (Mazziotta et al., 2021b), were included herein for comparison.

### MCPyV Linear Synthetic Peptides

The indirect enzyme-linked immunosorbent assay (ELISA) employed in this study to detect IgGs to MCPyV in sera from SA and VI was recently developed and validated (Mazziotta et al., 2021a,b). The immunoassay uses two linear synthetic peptides/mimotopes, known as MCPyV VP1 S and VP2 F (or S and F peptides) for detecting circulating IgGs against MCPyV in healthy adult and elderly individuals, as described (Mazziotta et al., 2021a,b). The peptides were synthesized using standard procedures and purchased from UFPeptides s.r.l., Ferrara, Italy. Amino acid (a.a.) sequences of VP1 S (24 a.a. residues) and VP2 F (25 a.a. residues) peptides are as follows:

VP1 S: NH_2_-NSPDLPTTSNWYTYTYDLQPKGSS-COOH and
VP2 F: NH_2_-SLSPTSRLQIQSNLVNLILNSRWVF-COOH.

### Indirect Enzyme-Linked Immunosorbent Assay

Indirect ELISA was performed as reported (Mazziotta et al., 2021a,b). S and F peptides, 5 μg each, were diluted in 100 μl of coating buffer 1X, pH 9.6 (Candor Bioscience, Wangen, Germany), which was used to coat each well of the immunological plates (Nunc-Immuno PolySorp, Thermo Fisher Scientific, Milan, Italy). The peptide-coated plates were incubated at 4°C for 16 h. Successively, immunological plates were rinsed three times with a washing buffer (Candor Bioscience, Wangen, Germany) to remove unbound peptides. For the blocking phase, 200 μl per well of blocking solution containing the casein and Tween detergent (Candor Bioscience, Wangen, Germany) was added to each well and incubated at 37°C for 90 min. Plates were washed three times with the washing buffer before serum samples were added. Each well was covered with 100 μl of serum samples diluted 1:20 in a low cross-buffer (Candor Bioscience, Wangen, Germany). Sera in each plate included (i) positive controls, that is, immune human sera derived from patients with MCPyV-positive MCC (Mazziotta et al., 2021b); (ii) negative controls, that is, three human MCPyV-negative sera (Mazziotta et al., 2021b); and (iii) sera from SA and VI under analysis. Immunological plates with sera were incubated at 37°C for 90 min. Each sample was analyzed in triplicate. Wells were washed three times, and then the secondary antibody was added to each sample. This solution consists of a goat anti-human IgG heavy (H)- and light (L)-chain-specific peroxidase conjugate (Calbiochem-Merck, Darmstadt, Germany) diluted 1:10,000 in a low cross-buffer. The solution was added to each well, whereas plates were incubated at room temperature (RT) for 90 min. After 90-min incubation, the plates were washed three times; then 100 μl of 2,2′-azino-bis-3-ethylbenzothiazoline-6-sulfonic acid (ABTS) solution (Sigma-Aldrich, Milan, Italy) was added to each well. Plates were incubated at RT for 45 min. ABTS reacted with the peroxidase enzyme to yield the color reaction. Finally, the plate was read with a spectrophotometer (Thermo Electron Corp., model Multiskan EX, Vantaa, Finland) at a wavelength (λ) of 405 nm. Color intensity in wells was determined by an optical density (OD) reading. The OD readings correspond to the amount of immune complexes formed by the specific antibodies binding to S and F synthetic peptides/mimotopes.

The cutoff for each S and F peptide was set in each indirect ELISA run, as the mean of the OD readings of negative control sera (*n* = 3) plus three SDs of mean (mean + 3 SDs) (Classen et al., 1987; Saraswati et al., 2019; Mazziotta et al., 2021a,b), as described previously for other ELISA methods (Meyer, 2001; Lardeux et al., 2016).

Immune serum samples were considered MCPyV positive when reacting to both S and F synthetic peptides, in three replica ELISA experiments carried out by three independent operators, without data variability.

### Statistical Analysis

Merkel cell polyomavirus seroprevalence rates were statistically analyzed applying a two-sided chi-square test (Tognon et al., 2020; Oton-Gonzalez et al., 2021). Values were analyzed using the D’Agostino Pearson normality test, and parametric and non-parametric tests were applied according to normal and non-normal variables, respectively, as reported (Nakagawa and Schielzeth, 2010; Rotondo et al., 2020a). In detail, ODs were analyzed using a one-way analysis of variance (ANOVA) and Kruskal–Wallis multiple comparison tests, according to normal and non-normal variables, respectively [OD medians, 95% confidence interval (CI)]. The Spearman correlation coefficient ***r*** was used to evaluate the OD concordance between S and F peptides. Statistical analyses were carried out using GraphPad Prism version 8.0 for Windows (GraphPad, La Jolla, CA, United States) (Rotondo et al., 2020b). A *p*-value < 0.05 was considered statistically significant (Mazzoni et al., 2020).

## Results

### Indirect Enzyme-Linked Immunosorbent Assay Reliability Assessment

**FIGURE 1:**
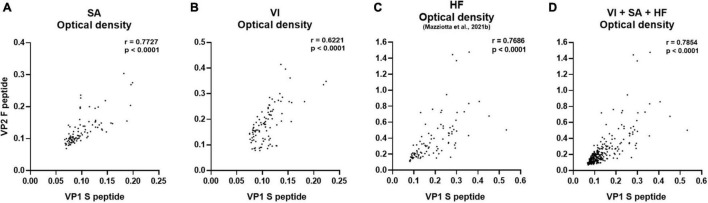
Correlation of OD values obtained using MCPyV VP1 S and VP2 F mimotopes. The concordance in ODs between VP1 S and VP2 F peptides was evaluated in sera from females who had SA (*n* = 94), the case; females undergoing VI (*n* = 96), the control; age-matched HF (*n* = 95); and the entire set of combined SA, VI, and HF sera (*n* = 285), using Spearman correlation analysis. A good correlation between VP1 S and VP2 F peptide was found in SA, VI, and HF sera considered alone, as well as in combined SA, VI, and HF sera with an *r* of 0.7727 and *p* < 0.0001 **(A)**, 0.6221 and *p* < 0.0001 **(B)**, 0.7686 and *p* < 0.0001 **(C)**, and 0.7854 and *p* < 0.0001 **(D)**, respectively.

In the first phase of this investigation, the reliability of our immunoassay was assessed in sera from female patients with SA and those who had undergone VI, by determining the OD concordance between MCPyV VP1 S and VP2 F peptides. OD concordance between S and F peptides was evaluated using the Spearman correlation analysis in SA (*n* = 94) and VI (*n* = 96) sera. In addition, immunological data from a group of age-matched HF (*n* = 95), which had previously been investigated in our laboratory for MCPyV serology (Mazziotta et al., 2021b), were included herein for statistical comparisons. OD concordance between S and F peptides was therefore analyzed in SA, VI, and HF sera considered alone, as well as in combination (*n* = 285). Results indicate a good degree of correlation between ODs for S and F peptides for the SA group, with a Spearman coefficient *r* of 0.7727 (*p* < 0.0001) (Figure 1A), and for the VI group, with an *r* of 0.6221 (*p* < 0.0001) (Figure 1B). A good correlation between S and F peptide ODs was also found in HF sera with an *r* of 0.7686 (*p* < 0.0001) (Figure 1C). In addition, when evaluating the combined SA, VI, and HF sera, a good concordance between S and F was determined with an *r* of 0.7854 (*p* < 0.0001) (Figure 1D). These data indicate that both peptides can be used simultaneously, therefore underlining that our assay is reliable in detecting anti-MCPyV IgGs in sera from SA and VI females.

### Detection of Serum IgG Antibodies Against MCPyV by Indirect Enzyme-Linked Immunosorbent Assay

Human sera, from SA (*n* = 94) and VI (*n* = 96) females, were analyzed by indirect ELISA for IgG Ab reactivity to MCPyV VP1 S and VP2 F peptides/mimotopes. The prevalence of anti-MCPyV IgGs was therefore determined in SA and VI groups. A statistically similar overall prevalence of 36.2% (34/94) and 43.6% (41/94) was obtained in SA sera, when tested with S and F peptides, respectively (*p* > 0.05) (Table 1). Comparable prevalence rates of 39.6% (38/96) and 47.9% (46/96) were obtained in VI sera reacting to S and F peptides, respectively (*p* > 0.05). With only a few exceptions, sera testing negative for S peptide did not react to F peptide and *vice versa*. In detail, 8.5% (8/94) of SA sera resulting negative for S peptide were positive for F peptide, while 1.1% (1/94) of sera tested F peptide negative, while being positive for S peptide. In VI, 10.4% (10/96) of sera tested F peptide positive were negative for S peptide, whereas 2.1% (2/96) of sera negative for F peptide were positive for S peptide.

**TABLE 1:** Seroprevalence of IgG antibodies reacting with Merkel cell polyomavirus VP1 S and VP2 F peptides in sera from females who experienced spontaneous abortion (SA) and females undergoing voluntary interruption (VI).

			Number of positive samples (%)		
Groups	Number of samples	Mean age (years) ± SD	VP1 S	VP2 F	VP1 S + VP2 F
SA	94	35 ± 6	34 (36.2)	41 (43.6)	33 (35.1)
VI	96	32 ± 7	38 (39.6)	46 (47.9)	36 (37.5)

*Serum samples were from SA (n = 94) and VI (n = 96) females. Statistical analyses were performed using the two-sided chi-square test. No statistical differences were detected between SA and VI groups (p > 0.05).*

In this study, sera were considered MCPyV positive when reacting with both mimotopes S and F, as previously reported (Mazziotta et al., 2021a,b).

The combined overall prevalence of IgGs against MCPyV, for both S and F peptides, was 35.1% (33/94) and 37.5% (36/96) in serum samples from SA and VI groups, respectively (*p* > 0.05).

### Serological Profiles of Serum IgG Antibodies Reacting to MCPyV

Serological profiles for IgG reactivity to MCPyV VP1 S and VP2 F peptides/mimotopes were analyzed, both for the single peptide and in combination. Immunological data were taken from sera belonging to SA (*n* = 94) and VI (*n* = 96) females. Results are reported as OD readings at λ 405 nm. The median [interquartile range (IQR)] ODs for S peptide and F peptide, alone and in combination, were then determined in SA and VI sera; then values were compared.

**FIGURE 2:**
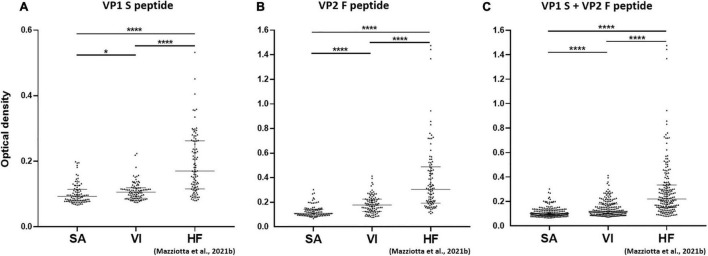
Serological profiles of serum antibody reactivity to MCPyV VP1 S **(A)**, VP2 F **(B)** peptides, and combined S and F peptides **(C)** in females who had SA, females who had undergone VI, and age-matched HF. Immunologic data are from SA (*n* = 94), VI (*n* = 96), and HF (*n* = 95) females, whereas data are reported as OD value readings at λ 405 nm for sera assayed in indirect ELISA. In the scatter dot plot, each dot represents the dispersion of ODs for each sample. The median is indicated by the line inside the scatter plot with the [interquartile range (IQR)] in SA and VI cohorts. **(A)** *p < 0.05, ****p < 0.0001; **(B)** ****p < 0.0001; **(C)** ****p < 0.0001.

Serologic profile analysis indicated that the median (IQR) OD values for S peptide resulted as 0.09 (0.08–0.11) and 0.11 (0.09–0.12) in SA and VI groups, respectively (Figure 2A). The median (IQR) OD values for F peptide resulted as 0.11 (0.1–0.14) and 0.18 (0.13–0.23) in SA and VI groups, respectively (Figure 2B). Lastly, the median (IQR) OD values for combined S and F peptides resulted as 0.1 (0.09–0.13) in SA and 0.12 (0.1–0.18) in VI groups (Figure 2C). The difference in OD levels between SA and VI groups was statistically significant for both S and F peptides considered alone, as well as upon combining ODs of the two peptides (*p* < 0.05, for S peptide; *p* < 0.0001, for both F peptide alone and combined S and F peptides) (Figure 2).

### Serum IgG Reactivity to MCPyV in Females Experiencing Spontaneous Abortion and Healthy Females Who Had Undergone Voluntary Interruption vs. Healthy Non-pregnant Females

In a second phase, MCPyV seroprevalence and serologic profiles in SA (*n* = 94) and VI (*n* = 96) females were compared to those obtained in sera from a group of age-matched HF (*n* = 95) females (*p* > 0.05), which had previously been investigated in our laboratory for MCPyV serology (Mazziotta et al., 2021b).

Merkel cell polyomavirus seroprevalence was found to be lower in both SA (35.1%, 33/94) and VI groups (37.5%, 36/96) than in the HF group (62.1%, 59/95) (*p* < 0.001). Serologic profile analysis indicated that S-peptide ODs were lower in both SA [*n* = 94, median (IQR), 0.09, 0.08–0.11] and VI groups (*n* = 96, 0.11, 0.09–0.12) than in HF (*n* = 95, 0.17, 0.12–0.26, *p* < 0.0001) (Figure 2A). Similarly, F-peptide ODs were lower in both SA (0.11, 0.1–0.14) and VI (0.18, 0.13–0.23) compared to the HF group (0.3, 0.19–0.49, *p* < 0.0001) (Figure 2B). Furthermore, lower ODs for combined S- and F-peptides were detected in both SA (0.1, 0.09–0.13) and VI sera (0.12, 0.1–0.18) compared to HF (0.22, 0.15–0.34, *p* < 0.0001) (Figure 2C).

## Discussion

The role of MCPyV in SA remains undetermined. Herein, MCPyV seroprevalence and serological profiles were investigated, for the first time, in sera from SA females using an indirect ELISA with two linear synthetic peptides/mimotopes, which mimic MCPyV VP antigens. The lack of immunological studies into the involvement of MCPyV in SA prompted us to apply our newly developed immunoassay (Mazziotta et al., 2021a,b). Sera were considered MCPyV positive when IgGs reacted to both S and F peptides (Mazziotta et al., 2021a,b).

Overall MCPyV-positive prevalence, resulted as 35.1% in SA, was comparable to the 37.5% determined in VI (*p* > 0.05). Our data indicate that both SA and VI female groups present circulating IgGs to MCPyV, while, at least in terms of seroprevalence, a relationship between MCPyV and SA might be unlikely.

The involvement of MCPyV infection in SA has been poorly investigated (Giakoumelou et al., 2016). Early findings indicate the presence of MCPyV DNA in uterine cervical cancer tissues (Imajoh et al., 2012), suggesting that, after infecting the uterus, this PyV could potentially participate as a SA risk factor. Nevertheless, few copies of MCPyV DNA and/or RNA molecules have been previously identified at low and similar prevalence in chorionic villi and PBMCs from SA and VI females (Tagliapietra et al., 2020), suggesting that MCPyV may not be involved in SA.

It is important to point out that SA females showed lower ODs compared to females who had undergone VI (*p* < 0.05), thus indicating a reduced anti-MCPyV IgG antibody response in females experiencing SA. In addition, both SA and VI females exhibited lower rates and ODs compared to those determined in age-matched, non-pregnant HF (62.1%), which had been investigated in our previous study conducted on healthy individuals (Mazziotta et al., 2021b). These findings indicate not only that serum IgG antibody response to MCPyV may decline in pregnancy but also that this decline is *de facto* more pronounced in females experiencing SA.

Notably, to the best of our knowledge, this is the first study reporting on MCPyV serology in SA. Only one previous study has reported on MCPyV serology in pregnancy, describing a rate of nearly 46% in a cohort of pregnant females (Sadeghi et al., 2010), a proportion similar to that obtained herein in our study/control groups. Regarding the healthy population, previous works have reported that a considerable fraction of adults, both males and females, is exposed to an asymptomatic MCPyV infection, with seroprevalence rates ranging from 60 to 80%, according to the study under consideration (Carter et al., 2009; Kean et al., 2009; Pastrana et al., 2009; Faust et al., 2011; Tolstov et al., 2011; Touzé et al., 2011; Viscidi et al., 2011; Coursaget et al., 2013; Van Der Meijden et al., 2013; Šroller et al., 2014; Zhang et al., 2014; Vahabpour et al., 2016; Kamminga et al., 2018; Csoboz et al., 2020; Zhou et al., 2020; Mazziotta et al., 2021a,b). Our immunological findings, alongside those previously reported, cumulatively indicate that a relatively reduced fraction of pregnant females carries anti-MCPyV IgGs compared to the healthy population, while SA females have a lower IgG antibody response to MCPyV.

The similar rate of immunological decrease determined in SA and VI females compared to non-pregnant females might be accounted for by the well-known immune adaptation/modulation process occurring in pregnancy (Longman and Johnson, 2007). Pregnant females present a unique state of immunity, as they develop a tolerance for the semi-allogeneic embryo/fetus, compared to non-pregnant females (Giakoumelou et al., 2016). In other words, despite recognizing the paternal antigens expressed by the embryo/fetus, the maternal immune system undergoes an adaptation to hamper her/his rejection (Mor and Cardenas, 2010). This adaptation is mainly, but not only, due to a decrease in the production and/or function of (i) lymphocyte T and natural killer cells, involved in the adaptive immune response (Longman and Johnson, 2007); and (ii) B cells, deputies in the production of antibodies (Medina et al., 1993). A transient immune modulation during pregnancy can therefore affect how SA and VI females respond to viral agents, making them more susceptible to viral infections (Giakoumelou et al., 2016), probably including MCPyV infection. The diminished serum IgG antibody response to MCPyV in both SA and VI females compared to non-pregnant females might be a reflection of the transient modulation in the immune function being experienced during pregnancy. The lower IgG antibody response to MCPyV determined in SA females might be linked to their more increased susceptibility to MCPyV infection. However, the relationship between this possible immunologic dysregulation and SA is unknown.

Conditions of physiological immune impairment in the host can favor a minor response to MCPyV as a result of antiviral surveillance decline (Ma and Brewer, 2014). This phenomenon can lead to an increase in MCPyV replication levels/activity or reinfection, ultimately leading to MCC (Ma and Brewer, 2014). A higher MCC rate has been determined in patients with immunocompromising conditions such as oncologic patients (Yu and Dasanu, 2016; Zheng et al., 2019) as well as in patients/individuals being pharmacologically treated for organ transplantation and/or autoimmune diseases (Lanoy and Engels, 2010; Boldorini et al., 2014; Clarke et al., 2015; Rotondo et al., 2017; Pietropaolo et al., 2020). Notably, MCC cases have also been identified in pregnant females (Chao et al., 1990; Paterson et al., 2003; Kukko et al., 2008; Tyler, 2020), thus supporting the view that a dysregulation in transient immune modulation during pregnancy can, at least in certain circumstances, favor an increase in MCPyV activity. It should be recalled that, despite being oncogenic, MCPyV is ubiquitous in humans, while establishing a lifelong and asymptomatic infection in healthy immunocompetent individuals. The decreased IgG antibody response to MCPyV might account for greater tolerance to viral infections experienced during pregnancy, which in turn might potentially lead to an increase in MCPyV replication levels/activity in SA females, as reported for other viral agents (Giakoumelou et al., 2016). Indeed, previous findings indicate that maternal immune tolerance impairment may result in SA or preeclampsia (Wang and Li, 2020).

How a diminished antibody response to MCPyV in SA females might negatively impact embryo development remains to be determined. Following an increase in MCPyV multiplication, this PyV might reach the embryo/fetus from the mother *via* vertical transmission, through the mother’s blood, as demonstrated for other viruses (Xu et al., 2015). As immune cells can allow viral crossing through placental barriers (Robbins and Bakardjiev, 2012; Cordeiro et al., 2015), a similar mechanism for MCPyV cannot be excluded. Notably, MCPyV DNA has been detected in white cells from pregnant females (Tagliapietra et al., 2020). An ascending transmission from the maternal reproductive system might be an alternative mechanism, as the uterine cervix may be prone to MCPyV infection (Imajoh et al., 2012). Likely, pregnancy may be affected following a maternal response to an MCPyV activity increase, even in the absence of a viral transmission, as found for other viruses (Mor and Cardenas, 2010; Racicot and Mor, 2017). Viral infections nearby placental barriers can trigger a mild inflammatory response represented by an increased cytokine production (Koga et al., 2009; Huang et al., 2014), promoting, in turn, an inflammatory response in the embryo/fetus, which can lead to pregnancy complications/abortion. A potential MCPyV activity increase may determine a mother’s inflammatory response, ultimately affecting pregnancy. Excluding chorionic villi and PBMCs (Tagliapietra et al., 2020), an increase in MCPyV activity, if present, due to a reduced anti-MCPyV immunological response in SA females might occur in a tissue type, which is currently unknown. Notably, the reservoir cell type of MCPyV is still unclear (Liu et al., 2019). Evidently, due to the lack of molecular data, further studies should be performed.

It is important to underline that MCPyV activity in terms of DNA replication and gene expression was not investigated herein. Therefore, the hypothesis that MCPyV activity might increase following a reduced antibody response to MCPyV, possibly making SA and VI females more susceptible to MCPyV infection, remains to be verified. Further studies evaluating the presence of viral DNA/RNA/proteins in key embryo development tissues including cord blood, amniotic fluids, cervical tissues, and fetal membranes should be conducted, as performed for other viruses (Giakoumelou et al., 2016). Since the basic immune conditions of SA, VI, and non-pregnant females might be different, determining the total IgG fraction, alongside IgGs to MCPyV, might help to better understand how pregnant females respond to MCPyV infection. Moreover, the prospective monitoring of the immunological status of pregnant females throughout the different gestation phases might be considered as an alternative investigative approach. These molecular and immunological experiments can be part of further investigations.

To avoid confounding variables, we excluded herein females who were positive for (i) congenital/acquired immunodeficiency syndromes or who were receiving immunosuppressive therapies; (ii) known causes of SA, i.e., genetic factors and anatomic/hormonal complications; and (iii) HIV, HBV, HCV, and syphilis infections. SA and VI females were similar in age (*p* > 0.05); samples were collected within 12 h from the abortion, while females were at a gestational age within the first 12 weeks.

In conclusion, our indirect ELISA proved to be reliable in identifying circulating IgGs reacting to MCPyV VP mimotopes in females experiencing SA and who had undergone VI. For the first time, IgG antibodies against oncogenic MCPyV were found in sera from SA females. Our results indicate that a reduced fraction of SA and VI females carries IgGs to MCPyV compared to healthy, non-pregnant females, while SA females presented a more pronounced decrease in IgG antibody response to MCPyV. Although yet to be determined, females experiencing abortive events might potentially present an increase in MCPyV multiplication events as a result of the immunological decline against MCPyV. Future investigations are needed to elucidate the relationship between MCPyV infection and SA.

## Data Availability Statement

The raw data supporting the conclusions of this article will be made available by the authors, without undue reservation.

## Ethics Statement

The studies involving human participants were reviewed and approved by the Ethical Committee of Ferrara, Italy, authorized the study (ID: 151078). The patients/participants provided their written informed consent to participate in this study.

## Author Contributions

JR: conceptualization. CM and JR: methodology. CM, JR, and GP: software. CM: formal analysis, resources, data curation, statistical analysis, and visualization. CM and GP: investigation. CM, MT, and JR: writing—original draft preparation. MT and FM: writing—review and editing. JR and MT: supervision. JR, MT, and FM: funding acquisition. All authors have read and agreed to the published version of the manuscript.

## Conflict of Interest

The authors declare that the research was conducted in the absence of any commercial or financial relationships that could be construed as a potential conflict of interest.

## Publisher’s Note

All claims expressed in this article are solely those of the authors and do not necessarily represent those of their affiliated organizations, or those of the publisher, the editors and the reviewers. Any product that may be evaluated in this article, or claim that may be made by its manufacturer, is not guaranteed or endorsed by the publisher.
